# Effects of posture on cerebellar evoked potentials (CEPs) following brief impulsive stimuli at the mastoid and trunk

**DOI:** 10.1007/s00221-022-06335-5

**Published:** 2022-03-04

**Authors:** Sendhil Govender, Neil P. M. Todd, James G. Colebatch

**Affiliations:** 1 Prince of Wales Clinical School, University of New South Wales, Randwick, Sydney, NSW 2052 Australia; 2 Neuroscience Research Australia, University of New South Wales, Randwick, Sydney, NSW 2052 Australia; 3 Institute of Neurological Sciences, Prince of Wales Hospital, Randwick, Sydney, NSW 2031 Australia

**Keywords:** Posture, Vestibular, Cerebellum, VsCEP, Evoked potentials

## Abstract

Recordings from over the posterior fossa following impulsive acceleration stimuli have shown short latency evoked potentials of presumed cerebellar origin. In this study, we investigated the effect of posture on these cerebellar evoked potentials (CEPs) and their relationship to postural reflexes recorded from the leg muscles evoked by the same stimuli. Nine healthy subjects were tested during lying (supine and prone), sitting and standing. Impulsive accelerations were applied at the mastoid and to truncal (both C7 and sternal) stimulation sites. The effect of vision, eyes open or closed, was investigated for all three stimuli. For the truncal stimuli, the effect of differing leaning conditions during standing was also recorded. CEP amplitudes were correlated for the three stimuli. For C7 stimulation during standing, both CEPs and postural reflexes scaled as the threat to postural stability increased. However, CEPs for all stimuli were present during lying, sitting and standing with amplitude and latency parameters mainly unaffected by posture or vision. In contrast, postural reflexes from the leg muscles were attenuated when not standing, with the effect being more marked for truncal stimuli. We conclude that CEPs evoked by axial and vestibular stimuli are not systematically gated by posture, in contrast to the reflex responses evoked by the same stimuli.

## Introduction

Short latency vestibular-evoked potentials in the cervical and ocular muscles following vestibular stimulation have been clearly delineated (e.g. Rosengren et al. 2010). Recently, evoked potentials recorded from over the posterior fossa of the scalp to impulsive head accelerations, an effective means of activating the otolith organs, have also been identified (Govender et al. 2020; Todd et al. 2018b, 2021). Studies have demonstrated these potentials to be modulated by visual context, lateralised corresponding to the direction of imposed head acceleration, and localised to sources in the cerebellum (Todd et al. 2018b, 2021). These potentials are thus referred to as cerebellar evoked potentials (CEPs). We have reported that these CEPs are maximal close to the midline at the base of the skull and are largest contralateral to the side being stimulated, for a positive polarity acceleration stimulus applied over the mastoid (Govender et al. 2020).

As well as being an effective vestibular stimulus, impulsive acceleration has also been utilised to investigate human postural control. Stimuli to the mastoid evoke short latency reflex responses in leg muscles similar to those seen with vestibular activation using galvanic stimulation (Laube et al. 2012). Brief perturbations of the upper trunk, using impulsive axial accelerations over the vertebra prominens (C7) and sternum also produce short latency postural responses in the leg muscles (Bötzel et al. 2001; Graus et al. 2013; Govender et al. 2015; Colebatch and Govender 2018). The evoked EMG responses are directed to the muscles most important for postural compensation and modulated by postural circumstances, the latter shown by the response becoming larger as the potential threat to postural stability increases. Both the mastoid-evoked and axially-evoked reflexes have characteristics of postural reflexes and, like galvanic-evoked responses, are presumed to relay through the brainstem (Britton et al. 1993). In a recent report, Todd et al. (2021) used both these forms of stimulation and showed localised changes in cerebellar activity occurring with them. The change in cerebellar activity appeared to correspond to changes in the evoked EMG response, raising the possibility that the output of the cerebellum might modulate these postural reflexes.

Postural reflexes share the characteristic feature that they are strongly modulated by postural conditions, in particular, whether subjects are standing or not. When not standing, postural reflexes are severely attenuated (Britton et al. 1993; Govender et al. 2015). Given that both our stimuli evoke postural reflexes, which we have proposed are likely to be mediated by spino-bulbar-spinal pathways (Colebatch and Govender 2018), it is pertinent to investigate whether the responses we have previously shown localised to the cerebellum are also modified by posture. Parallel alterations of the cerebellar evoked responses (CEPs) would suggest that the postural modulation might act on afferent input and indicate a specific role for the cerebellum. Failure to show such changes would suggest the changes with posture mainly occurred on the efferent limb of the postural reflex. We have, therefore, examined the effects of a variety of postural conditions on the associated CEPs and evoked EMG responses in the leg muscles.

## Methodology

### Participants

Nine normal subjects (mean: 34 years; age range: 18–65 years; 7 male, 2 female) with no prior history of vestibular, hearing or neurological impairment were recruited from staff and students at the Prince of Wales Hospital and University of New South Wales, Sydney, Australia. Informed consent was obtained prior to experimentation and in accordance with the Declaration of Helenski. The study was approved by the local ethics committee (South Eastern Sydney Local Health District Human Research Ethics Committee; HREC 18/071).

### Impulsive acceleration stimuli

The stimulus waveform consisted of impulsive accelerations (a 3rd order gamma waveform with a 4 ms rise time) generated using a laboratory interface (CED power1401,Cambridge Electronic Design, Cambridge, UK), a power amplifier (model 2718, Brűel & Kjær, Denmark) and customised software. The stimulus was designed to produce an approximately incompressive whole head movement and minimise any elastic bone conduction component (Todd et al. 2008a). It was delivered using a hand-held mini-shaker device (model 4810, Brüel and Kjaer P/L, Denmark) with an attached cylindrical perspex rod (diameter: 2.5 cm, length 9.2 cm). Three stimulation sites were used; the left mastoid process, the vertebra prominens (C7) and the sternal manubrium. Sternal and C7 stimuli were both used because they have opposite postural effects. The initial polarity of the stimulus was positive (movement of the rod towards the subject) for all stimulation sites, delivered at a rate of ~ 3 Hz and at a fixed intensity of 20 V peak [~ 14 N peak force level (FL)].

### EEG/ECeG and EMG recordings

**Fig. 1:**
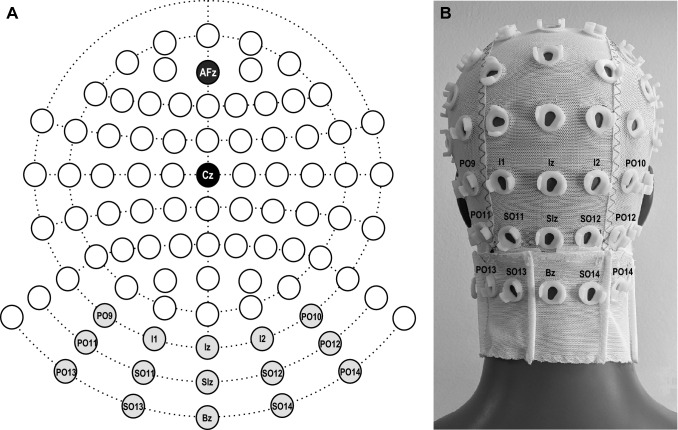
**A** Custom EASYCAP EEG/ECeG layout showing the selected three posterior rows of 5 electrodes (light grey), and reference (AFz; dark grey) and earth electrodes (Cz; black). **B** Posterior view of the custom EASYCAP setup with the selected recording electrodes shown over the posterior fossa and neck

EEG/ECeG (electrocerebellogram) was recorded using a 10–10 cerebellar-extended cap (EASYCAP GmbH, Germany) with a subset of electrodes chosen based on our previous studies (Govender et al 2020; Todd et al. 2021). Fifteen recording locations were used and consisted of three rows of five electrodes over the posterior of the scalp and neck (see Fig. 1—top row: PO9, I1, Iz, I2 and PO10; middle row: PO11, SO11, SIz, SO12 and PO12; bottom row: PO13, SO13, Bz, SO14 and PO14). The ground electrode was positioned at Cz and a reference electrode at AFz. EEG/ECeG signals were amplified (20,000×) and filtered (0.5 Hz to 3 kHz) using two D360 amplifiers (Digitimer Ltd, Welwyn Garden City, UK). A 50 Hz notch filter was used during recordings.

EMG recordings were made bilaterally from over the soleus and tibialis anterior (TA) muscles using surface adhesive electrodes (Cleartrace 1700-030, Conmed Corp., USA). Active electrodes were positioned 1–2 cm above the musculotendinous junction for soleus and 1–2 cm lateral to the tibia for TA with reference electrodes 2 cm below the active electrodes. A ground electrode was placed on the midpoint of the right lower leg. EMG signals were amplified (2500×) and filtered (8 Hz to 1.6 kHz) using AA6 Mk III amplifiers (Medelec Ltd, Old Woking, Surrey UK).

EEG/ECeG and EMG activity were recorded using Signal software (version 6.02, Cambridge Electronic Design, Cambridge, UK) and a Micro1401 (Cambridge Electronic Design, Cambridge, UK). Recordings were sampled at 10 kHz from 50 ms prior to stimulus onset to 250 ms after. EMG was rectified and averaged offline.

### Experimental procedure

Fifteen conditions were recorded across four different postures: supine, prone, sitting upright without support and standing (Table 1). Supine and prone positions allowed for only a single truncal site to be accessible. To ensure adequate tonic levels of activation in the leg muscles when supine, prone and sitting, subjects were asked to maintain plantar flexion of the feet during C7 and mastoid stimulation and to maintain dorsiflexion of the feet during sternal stimulation. The mastoid was stimulated in both supine and prone positions while C7 and sternal stimulation were applied in only the prone and supine positions respectively. While sitting upright without support, subjects were stimulated at all three sites. During standing, the mastoid was stimulated during normal upright stance, C7 stimulation during anterior lean and sternal stimulation during posterior lean, with both eyes open and eyes closed conditions being tested. Sternal stimulation during posterior lean increases its reflex effectiveness, and similarly for anterior lean and C7 stimulation (Govender et al. 2015). Additional recordings were also made during C7 stimulation with posterior lean and sternal stimulation with anterior lean, conditions in which the perturbations are less threatening to postural stability and evoke smaller responses. The order of conditions was randomised between subjects.

**Table 1:** The list of experimental conditions tested

Posture	Stimulation site	Tonic activation of leg muscles
Supine (EO)	Mastoid	Plantar flexion
	Sternum	Dorsiflexion
Prone (EO)	Mastoid	Plantar flexion
	C7	Plantar flexion
Sitting upright without support (EO)	Mastoid	Plantar flexion
	Sternum	Dorsiflexion
	C7	Plantar flexion

Posture	Stimulation site	Leaning posture
Standing (EO)	Mastoid	Neutral
	Sternum	Anterior
	Sternum	Posterior
	C7	Anterior
	C7	Posterior
Standing (EC)	Mastoid	Neutral
	Sternum	Posterior
	C7	Anterior

*EO* eyes open, *EC* eyes closed

### Data analysis

Evoked EEG/ECeG response amplitudes and latencies were measured at Iz for C7 and sternal (midline) stimulation and PO10 for vestibular (lateralised) stimulation, based upon our previous studies (Govender et al. 2020; Todd et al. 2021). Because the evoked responses are more marked for high frequencies (Todd et al. 2018a, 2021), RMS (root mean square) averaging with high pass filtering (160 Hz) was used to measure both tonic activity levels and the period of suppression. The period of suppression (inhibition) was quantified as the percentage decrease from baseline levels. Evoked EMG response amplitudes were normalised against their baseline levels and latencies were measured at the onset and end of the initial peak, with mean values taken as the average of the right and left sides. Baseline rectified EMG levels were averaged between sides. Separate ANOVAs were carried out for each stimulation site using posture and peak as factors (C7—prone, sitting and standing; Mastoid—supine, prone, sitting and standing; Sternum– supine, sitting and standing). ANOVAs were also conducted using vision (eyes open vs eyes closed) and response peak as factors for all three stimuli and lean (anterior and posterior) and response peak as factors for C7 and sternal stimulation. The Greenhouse–Geisser correction was used to correct for violation of the assumption of sphericity. Correlations compared the amplitudes of the various peaks between the modalities between subjects. Correlations were also performed using evoked response amplitudes against the magnitude of suppression of EEG/ECeG activity for each modality. For correlations, the level of significance was set at *P* = 0.01 due to the number of comparisons. Mean ± SD are reported in the text and tables, and mean ± SEM values are shown in figures.

## Results

### C7 stimulation

**Fig. 2:**
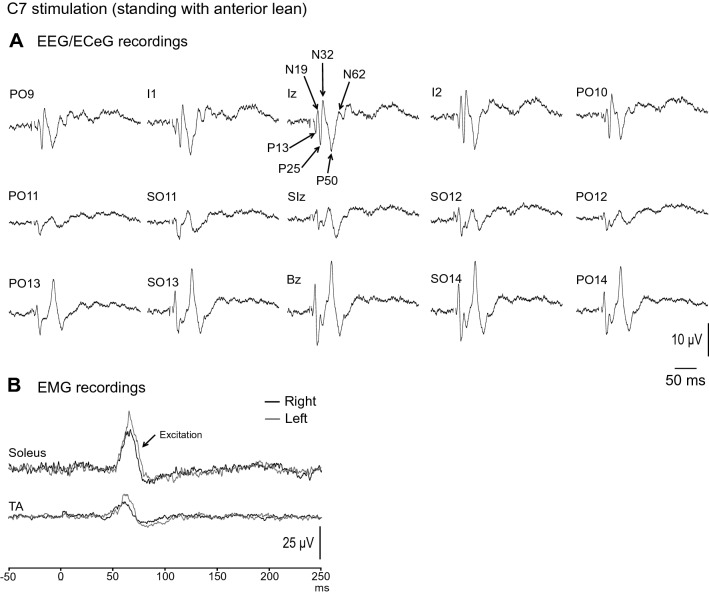
Grand mean EEG/ECeG and EMG recordings to C7 stimulation during standing with anterior lean and eyes open. **A** The evoked EEG/ECeG response was characterised by a series of positive and negative waves (P13, N19, P25, N32, P50 and N62) and was present mainly in the Iz row of electrodes. **B** The evoked EMG response was characterised by an excitation recorded in the soleus muscles, consistent with the agonist muscle acting to counter the postural threat of an anterior perturbation. A smaller excitation can also be observed in the tibialis anterior (TA) muscle group. Stimulus artefact has been clipped to improve response clarity

Grand mean evoked EEG/ECeG and EMG responses to C7 stimulation during standing with anterior lean and eyes open are shown in Fig. 2. Similar to our previous report (Todd et al. 2021), the evoked response to C7 impulsive stimulation consisted of a series of 3 pairs of positive–negative waves (P13/N19/P25/N32/P50/N62), named by their average latencies. These were prominent at Iz and the electrodes lateral to it (i.e. PO9, I1, I2 and PO10) A phase inversion of the evoked response was seen at electrodes positioned lower over the neck (PO13, SO13, Bz, SO14 and PO14; Fig. 2A). In the leg muscles, an excitatory evoked EMG response was recorded bilaterally from soleus with a mean corrected amplitude of 46 ± 26%, and onset and end latencies of 56.4 ± 2.5 ms and 76.2 ± 4.9 ms respectively (Fig. 2B).

**Fig. 3:**
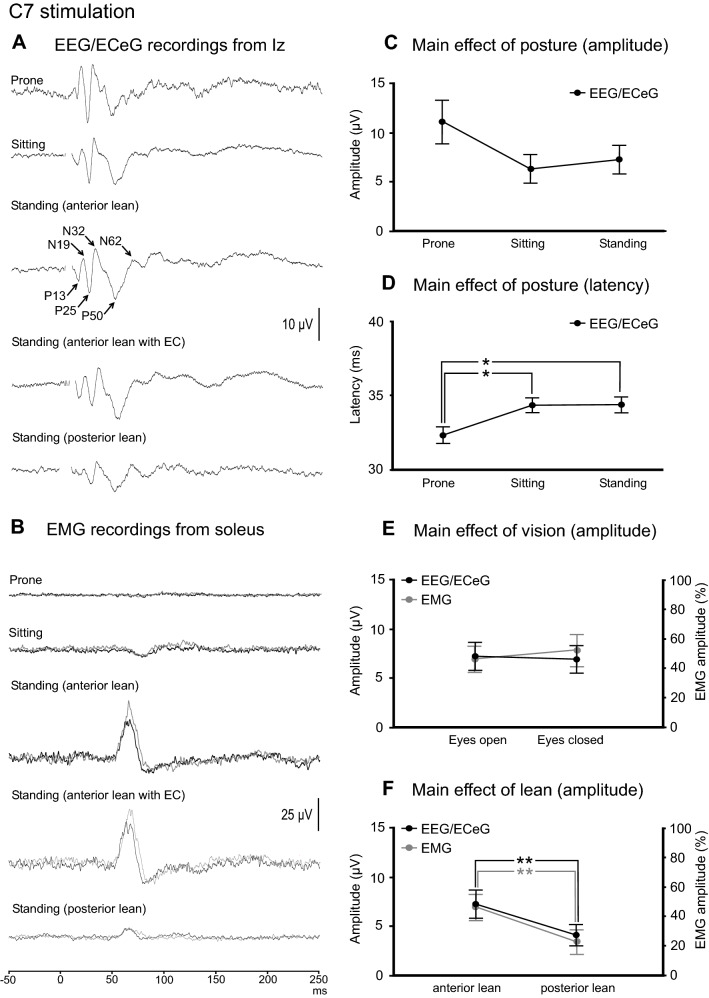
**A** Grand mean recordings from Iz show the evoked response to be present across the conditions tested, whereas the evoked EMG response in soleus was present only during standing (**B**). While the main effects of posture were not significant at Iz for amplitude (**C**) or latency (**D**), the prone position tended to produce larger and earlier responses. Vision did not affect EEG/ECeG or EMG evoked responses (**E**). For both EEG/ECeG and EMG evoked responses, anterior lean produced larger amplitudes than posterior lean (**F**). Baseline rectified EMG levels for soleus were 17.4 (prone), 30.5 (sitting), 54.7, 54.0 (standing, anterior lean, eyes open and closed) and 24.6 µV (posterior lean). *P < 0.05, **P < 0.01

Mean amplitudes, latencies, ranges and prevalence for the evoked response peaks recorded at Iz are given in Table 2 for C7 stimulation. The P25, N32 and P50 peaks were generally more frequently present than the P13, N19 and N62 peaks. With averaging, the evoked response at Iz was present across all the postural conditions tested (Fig. 3A). The evoked EMG response was present only during standing and was abolished when in the prone and sitting positions (Fig. 3B). Baseline rectified EMG levels were 54.7 µV (soleus) and 24.6 µV (TA).

**Table 2:** Mean amplitudes, latencies, ranges and prevalence of evoked response peaks recorded at Iz following C7 stimulation

Condition		Peaks					
		P13	N19	P25	N32	P50	N62
Prone	Amplitude (µV)	5.6 (7.2)	7.0 (6.1)	16.4 (16.9)	13.5 (10.2)	17.4 (11.2)	6.5 (6.4)
	Latency (ms)	13.4 (0.7)	17.2 (1.3)	25.3 (2.2)	29.8 (1.9)	47.9 (7.4)	60.3 (5.0)
	Range (µV)	[0–17]	[0–15.5]	[0–48.0]	[0–29.9]	[0–33.4]	[0–16.7]
	Prevalence (%)	44	67	89	89	89	67
Sitting	Amplitude (µV)	3.0 (2.9)	3.9 (4.3)	9.1 (13.3)	6.8 (7.0)	11.0 (4.2)	4.0 (4.3)
	Latency (ms)	13.1 (1.7)	19.1 (2.1)	25.2 (1.4)	31.6 (3.1)	51.6 (5.9)	65.4 (2.3)
	Range (µV)	[0–8.3]	[0–11.9]	[1.0–43.6]	[0–20.1]	[5.3–19.0]	[0–11.1]
	Prevalence (%)	67	67	100	89	100	78
Standing (anterior lean)	Amplitude (µV)	4.0 (4.9)	4.4 (5.7)	10.2 (9.6)	7.1 (6.2)	13.3 (6.5)	4.7 (6.0)
	Latency (ms)	14.2 (1.2)	19.7 (0.8)	25.1 (2.0)	31.1 (1.4)	52.2 (5.1)	64.0 (2.6)
	Range (µV)	[0–14.5]	[0–18.4]	[2.9–31.5]	[1.5–22.7]	[5.4–22.8]	[0–17.1]
	Prevalence (%)	67	67	100	100	100	56
Standing (anterior lean, EC)	Amplitude (µV)	4.2 (5.7)	4.7 (5.5)	9.9 (12.3)	6.3 (6.1)	14.3 (6.4)	2.5 (5.6)
	Latency (ms)	13.5 (0.8)	19.7 (1.8)	26.0 (1.6)	32.3 (2.1)	53.1 (4.9)	65.3 (2.0)
	Range (µV)	[0–17.2]	[0–15.8]	[0–41.1]	[1.3–20.3]	[7.9–26.4]	[0–16.5]
	Prevalence (%)	67%	67	89	100	100	22
Standing (posterior lean)	Amplitude (µV)	2.4 (4.1)	0.8 (1.5)	5.2 (5.2)	3.6 (3.9)	9.5 (5.8)	3.2 (4.7)
	Latency (ms)	14.4 (2.2)	20.5 (0.1)	26.8 (2.1)	32.4 (1.5)	50.1 (5.1)	64.5 (0.8)
	Range (µV)	[0–12.1]	[0–3.8]	[0–16.3]	[0–10.3]	[0–16.9]	[0–12.0]
	Prevalence (%)	44	22	78	67	89	44

Mean (SD)

*EC* eyes closed

The amplitude of the evoked responses at Iz were unaffected overall by posture (*F*_(2,16)_ = 3.4, *P* = 0.096), although the prone position did tend to produce larger recordings (Fig. 3C). Latencies were slightly earlier in the prone position (Fig. 3D; overall means; prone = 32.3 ms, sitting & standing = 34.4 ms; *F*_(2,113)_ = 4.7, *P* = 0.011). Vision did not affect amplitude (Fig. 3E; *F*_(1,8)_ = 0.2, *P* = 0.664) or latency (*F*_(1,72)_ = 0.8, *P* = 0.376). Anterior lean produced larger amplitudes compared to posterior lean (Fig. 3F—black line; overall means; anterior lean = 7.3 µV, posterior lean = 4.1 µV; *F*_(1,8)_ = 12.8, *P* = 0.007) and this was also observed for the evoked EMG response (Fig. 3F—grey line; overall means; anterior lean = 46 ± 26%, posterior lean = 23 ± 24%; *t*_(8)_ = 3.4, *P* = 0.009). EMG responses were unaffected by vision [*t*_(8)_ = 1.0 (amplitude) = 0.334; *t*_(8)_ = 0.4 and 1.3 (onset and end latency), *P* = 0.691 amd 0.244].

### Mastoid stimulation

**Fig. 4:**
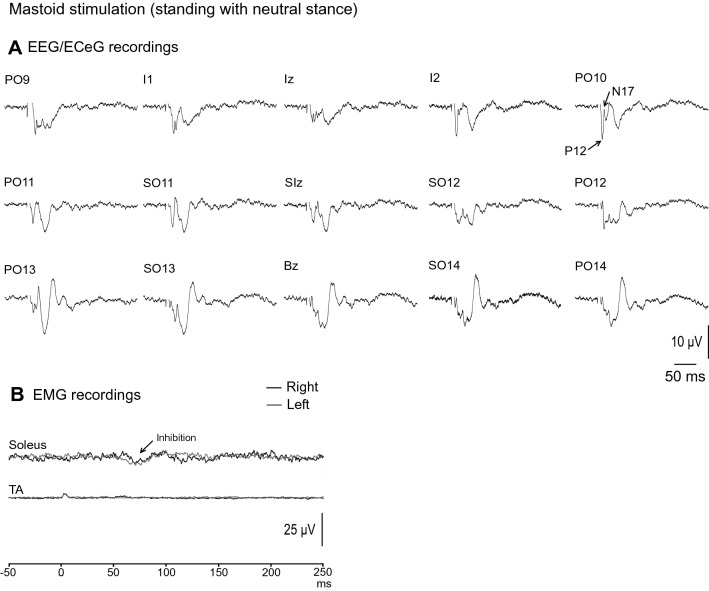
Grand mean EEG/ECeG and EMG recordings to mastoid stimulation during standing in neutral stance and eyes open. **A** Left mastoid stimulation produced a lateralised response characterised by a P12-N17 short latency response on the contralateral side at the PO10 location. **B** Only a small inhibition was observed in the soleus muscles as the head was not rotated during the recordings. Baseline rectified EMG levels were 32.0 µV (soleus) and 9.0 µV (TA)

EEG/ECeG and EMG responses to mastoid stimulation during neutral stance and eyes open are shown in Fig. 4. As previously reported (Govender et al. 2020), the evoked response consisted of a biphasic P12/N17 response, and was largest over the PO10 location (Fig. 4A). The head was not rotated to avoid vestibular-evoked myogenic potential (VEMP) contamination of the EEG/ECeG response. As a result of the head being straight rather than rotated, only a small inhibition was observed in the soleus muscles during standing, with mean corrected amplitude of 9 ± 7%. The onset and end latencies were 56.5 ± 4.4 ms and 87.0 ± 8.9 ms respectively in the eyes open condition (Fig. 4B).

**Fig. 5:**
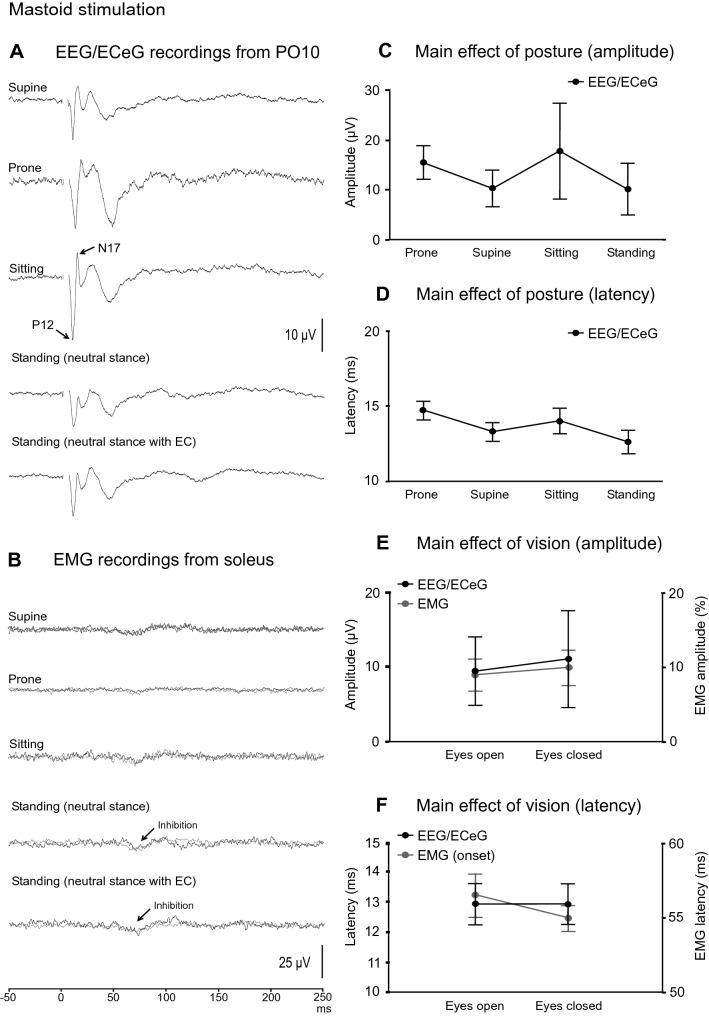
**A** Grand mean recordings from PO10 show the P12-N17 evoked response present across the conditions tested with mastoid stimulation. The evoked EMG response in soleus was usually absent but a small inhibition was observed in most subjects during standing (**B**). The evoked EEG/ECeG response was more variable during sitting but the main effect of posture was not significant overall (**C**). Latency was unaffected by posture (**D**) and neither EEG/ECeG nor EMG were affected by vision (**E**, **F**). Baseline rectified EMG levels were 32.3 (supine), 22.6 (prone), 39.1 (sitting), 32.0 and 32.2 µV (neutral stance, eyes open and closed)

Mean amplitudes, latencies, ranges and prevalence for the evoked response peaks recorded at PO10 are given in Table 3. With averaging, potentials were present at PO10 for all the conditions tested (Fig. 5A) and the P12 and N17 potentials were present in all subjects when prone and in all but one when supine. The morphology of the evoked waveform did change with the N17 peak being less frequent during the standing conditions (56% prevalence) compared to the P12 peak (100% prevalence). EMG evoked responses were usually absent for mastoid stimulation with a small inhibitory response for most subjects during standing (Fig. 5B).

**Table 3:** Mean amplitudes, latencies, ranges and prevalence of evoked response peaks recorded at PO10 following left mastoid stimulation

Condition		Peaks	
		P12	N17
Supine	Amplitude (µV)	14.6 (16.4)	9.6 (9.5)
	Latency (ms)	11.5 (1.5)	15.2 (1.6)
	Range (µV)	[0–47.2]	[0–33.0]
	Prevalence (%)	89	89
Prone	Amplitude (µV)	19.3 (13.5)	11.5 (7.7)
	Latency (ms)	13.0 (1.6)	16.4 (1.9)
	Range (µV)	[4.5–45.5]	[1.9–26.4]
	Prevalence (%)	100	100
Sitting	Amplitude (µV)	22.7 (29.3)	12.7 (22.2)
	Latency (ms)	11.8 (1.7)	16.4 (2.5)
	Range (µV)	[0–91.6]	[0–67.6]
	Prevalence (%)	78	78
Standing (neutral stance)	Amplitude (µV)	13.4 (16.1)	5.6 (11.9)
	Latency (ms)	11.5 (2.5)	14.4 (2.9)
	Range (µV)	[4.9–54.6]	[0–36.6]
	Prevalence (%)	100	56
Standing (neutral stance, EC)	Amplitude (µV)	14.9 (21.8)	7.2 (17.1)
	Latency (ms)	11.4 (2.4)	14.5 (2.7)
	Range (µV)	[4.7–72.6]	[0–52.4]
	Prevalence (%)	100	56

Mean (SD)

*EC* eyes closed

Overall, posture did not affect amplitudes (Fig. 5C; *F*_(3,21)_ = 0.7, *P* = 0.450), nor latencies (Fig. 5D; *F*_(3,53)_ = 2.3, *P* = 0.09), although there was a trend for slightly later responses in the prone position (overall means; supine: 13.3 ms, prone: 14.7 ms, sitting: 14.1 ms, standing: 12.6 ms). Vision did not affect amplitudes at PO10 (Fig. 5E; *F*_(1,8)_ = 0.6, *P* = 0.445) nor latency parameters (Fig. 5F; *F*_(1,26)_ = 0.01, *P* = 0.921). EMG responses were unaffected by vision [*t*_(8)_ = 0.3 (amplitude), *P* = 0.737; *t*_(8)_ = 1.1 and 0.5 (onset and end latency), *P* = 0.288 and 0.648].

### Sternal stimulation

**Fig. 6:**
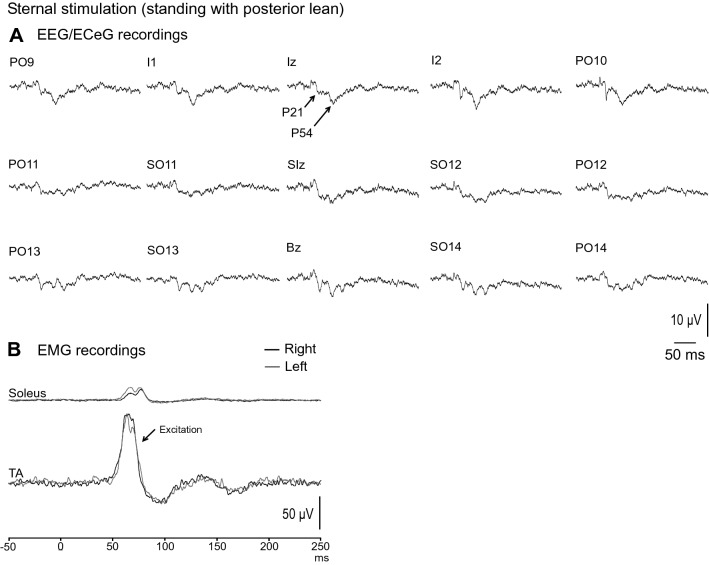
Grand mean EEG/ECeG and EMG recordings to sternal stimulation during standing with posterior lean and eyes open. **A** EEG/ECeG evoked responses were less prominent for sternal stimulation compared to C7 and mastoid stimulation, with the most consistent potentials being the P21 and P54 peaks. **B** Despite sternal stimulation being less effective in evoking EEG/ECeG responses, large and robust EMG responses were recorded from the tibialis anterior (TA) muscles. Baseline rectified EMG levels were 17.0 (soleus) and 73.8 µV (TA)

Sternal stimulation was the least effective in evoking EEG/ECeG responses (Fig. 6A). The most prominent and consistent waves recorded at Iz were the positive peaks at mean latencies of 21 (P21) and 54 ms (P54). In contrast, large EMG evoked responses were recorded bilaterally in the TA muscles, consistent with the muscle acting as an agonist to counter the threat to postural stability (Fig. 6B). During posterior lean with eyes open, the amplitude of the evoked EMG response was 89 ± 35% with onset and end latencies of 55.0 ± 4.0 ms and 79.6 ± 5.2 ms respectively.

**Fig. 7:**
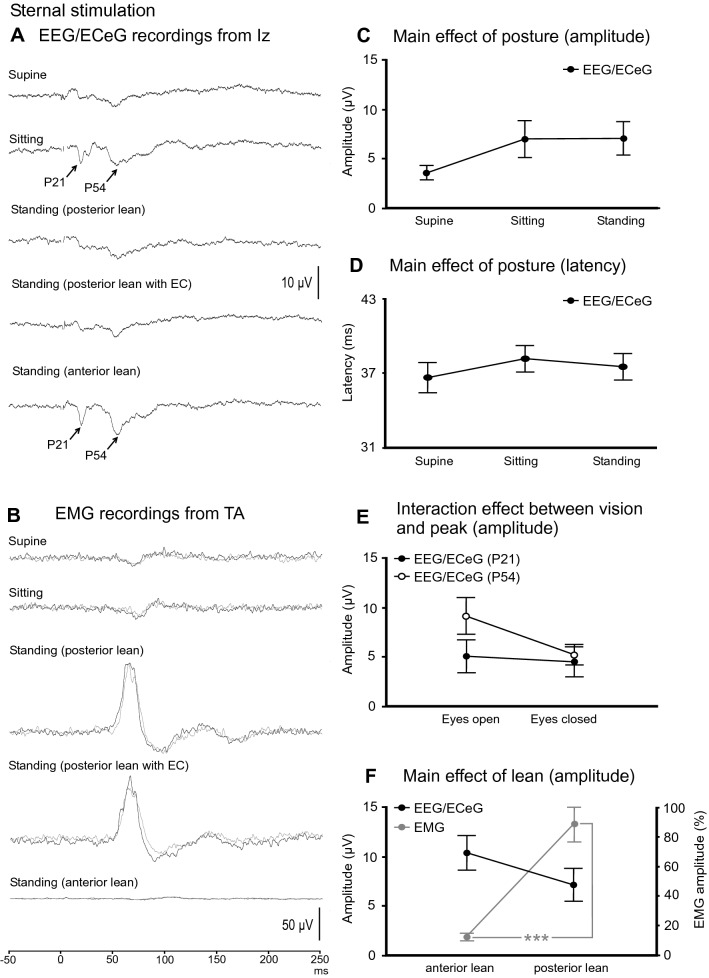
**A** Grand mean recordings from Iz during sternal stimulation show the P21 and P54 peaks to be less prominent and not well formed across the conditions tested. The evoked EMG response in tibialis anterior (TA) was attenuated in the supine and sitting conditions as well as during standing with anterior lean, which does not pose a threat to postural stability. The evoked response at Iz tended to be smaller in the supine position (**C**) while latency was unaffected by posture (**D**). The P54 peak was more affected by vision than the P21 peak, with smaller amplitudes during eye closure (**E**). Anterior lean tended to produce larger EEG/ECeG responses than posterior lean whereas EMG responses were significantly larger during posterior lean (**F**). Baseline rectified EMG levels for TA were 69.1 (supine), 82.4 (sitting), 73.8, 64.8 (standing, posterior lean, eyes open and closed) and 11.2 µV (standing, anterior lean). ***P < 0.001

Mean amplitudes, latencies, ranges and prevalence for the evoked response following sternal stimulation are given in Table 4. The P21 (67–89% prevalence) and P54 (78–100% prevalence) peaks were more evident during sitting and during anterior lean (Fig. 7A). In contrast, the evoked EMG response was attenuated during the supine, sitting and standing with anterior lean conditions (Fig. 7B).

**Table 4:** Mean amplitudes, latencies, ranges and prevalence of evoked response peaks recorded at Iz following sternal stimulation

Condition		Peaks	
		P21	P54
Supine	Amplitude (µV)	3.1 (2.8)	4.1 (2.7)
	Latency (ms)	21.6 (3.5)	51.7 (3.8)
	Range (µV)	[0 – 7.2]	[0 – 7.6]
	Prevalence (%)	67	78
Sitting	Amplitude (µV)	6.0 (6.2)	8.1 (5.4)
	Latency (ms)	20.8 (2.4)	55.5 (5.6)
	Range (µV)	[0 – 20.5]	[3 – 21.2]
	Prevalence (%)	89	100
Standing (posterior lean)	Amplitude (µV)	5.1 (5.0)	9.2 (5.6)
	Latency (ms)	20.8 (2.5)	54.2 (6.2)
	Range (µV)	[0 – 14.9]	[3 – 22.3]
	Prevalence (%)	89	100
Standing (posterior lean, EC)	Amplitude (µV)	4.5 (4.7)	5.2 (3.1)
	Latency (ms)	22.3 (3.0)	53.7 (4.2)
	Range (µV)	[0 – 15.0]	[0 – 10.4]
	Prevalence (%)	78	89
Standing (anterior lean)	Amplitude (µV)	8.3 (6.8)	12.5 (5.2)
	Latency (ms)	21.2 (1.5)	55.5 (5.3)
	Range (µV)	[0 – 23.3]	[5.4 – 21.3]
	Prevalence (%)	89	100

Mean (SD)

*EC* eyes closed

While posture did not affect the evoked response at Iz overall for sternal stimulation (*F*_(2,16)_ = 2.7, *P* = 0.098), there was a trend for smaller responses in the supine position (Fig. 7C; overall means; supine: 3.6 µV, sitting: 7.0 µV, standing: 7.1 µV). Latency was unaffected by posture (Fig. 7D; *F*_(2,41)_ = 0.4, *P* = 0.661). The later P54 peak was more affected by vision than the initial P21 peak, with P54 amplitude decreasing during eye closure (Fig. 7E; interaction effect between vision and peak; *F*_(1,8)_ = 6.2, *P* = 0.037). Latency was unaffected by vision (*F*_(1,28)_ = 0.1, *P* = 0.738) or leaning posture (*F*_(1,30)_ = 0.3, *P* = 0.586). Anterior lean tended to produce larger responses than posterior lean (Fig. 7F – black line; overall means; anterior lean: 10.4 µV, posterior lean: 7.1 µV; *F*_(1,8)_ = 5.1, *P* = 0.053).

EMG response amplitudes (*t*_(8)_ = 1.3, P = 0.241) and latencies (*t*_(8)_ = 0.5 & 1.5 (onset and end latency), *P* = 0.600 and 0.169) were unaffected by vision. In contrast to the evoked EEG/ECeG response, posterior lean produced larger EMG amplitudes than anterior lean (Fig. 7F—grey line; anterior lean: 13 ± 7%, posterior lean: 89 ± 35%; *t*_(8)_ = 6.3, *P* < 0.001).

### Correlation of peak amplitudes between stimulus modalities

The C7-evoked P25 peak amplitude was positively correlated between subjects with the mastoid-evoked P12 (*r* = 0.55, *P* = 0.002) and the sternally evoked P21 peaks (*r* = 0.51, *P* < 0.001). The C7-evoked N62 peak showed a positive correlation with the mastoid-evoked P12 (*r* = 0.58, *P* < 0.001) and N17 peak amplitudes (*r* = 0.53, *P* < 0.001). Similarly, the sternally evoked P21 peak was correlated with both mastoid-evoked amplitudes (P12; *r* = 0.59, *P* < 0.001 and N17; *r* = 0.45, *P* = 0.002).

### RMS averaging

**Fig. 8:**
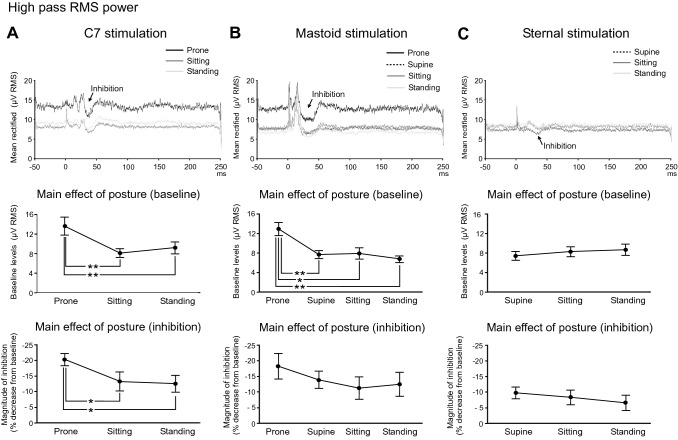
Grand mean averages of high pass RMS power (top row) measured from the Iz (**A** and **C**) or PO10 (**B**) electrodes and the effects of posture. Baseline levels (middle row) and the magnitude of inhibition of tonic activity (bottom row) are shown for C7 (**A**), mastoid (**B**) and sternal stimulation (**C**). Grand means revealed differences in baseline RMS levels between postures with C7 and mastoid stimulation associated with higher levels in the prone condition. The prominent inhibitory response was also modulated by posture for C7 stimulation. The magnitude of inhibition was significantly greater in the prone condition for C7 stimulation. *P < 0.05, **P < 0.01

High pass RMS averages were made for Iz activity for C7/sternal stimulation and PO10 activity for mastoid stimulation across the postural conditions tested. This revealed changes both in baseline levels and post stimulus suppression. There was an increase in baseline RMS levels for lying prone but not supine (Fig. 8 and Table 5). RMS levels were higher when tested prone for both C7 (Fig. 8A; *F*_(2,16)_ = 9.1, *P* = 0.002) and mastoid stimulation (Fig. 8B; *F*_(3,21)_ = 11.4, *P* < 0.001). The higher tonic level was associated with a larger evoked response for both C7 and mastoid stimulation. No such increase was present for lying supine, thus RMS levels for sternal stimulation were unaffected by posture (Fig. 8C; *F*_(2,16)_ = 1.1, *P* = 0.353). The period of inhibition following C7 stimulation was larger in the prone position (*F*_(2,16)_ = 5.7, *P* = 0.013). For mastoid stimulation, the prone condition produced the largest inhibitory response, but the overall effect of posture was not significant (*F*_(3,21)_ = 1.2, *P* = 0.327). The smaller inhibition observed during sternal stimulation was unaffected by posture (*F*_(2,16)_ = 0.6, *P* = 0.566).

**Table 5:** Mean baseline RMS levels and magnitude of inhibition for each postural condition and stimulus site

	Condition			
	Prone	Supine	Sitting	Standing
C7 (Iz)				
Baseline RMS (µV)	13.6 ± 5.6	–	8.1 ± 2.6	9.2 ± 3.6
Magnitude of inhibition (%)	− 20 ± 6	–	− 13 ± 9	− 13 ± 8
Mastoid (PO10)				
Baseline RMS (µV)	12.9 ± 3.9	7.6 ± 2.4	7.9 ± 3.5	6.8 ± 2.0
Magnitude of inhibition (%)	− 18 ± 12	− 14 ± 8	− 11 ± 11	− 12 ± 12
Sternal (Iz)				
Baseline RMS (µV)	–	7.4 ± 2.7	8.3 ± 3.0	8.7 ± 3.5
Magnitude of inhibition (%)	–	− 10 ± 6	− 8 ± 7	− 7 ± 7

The recording electrode studied is given in brackets for each stimulus condition

For C7 stimulation, the magnitude of the period of inhibition was correlated with the P13 (*r* = 0.55, *P* = 0.003), P19 (*r* = 0.59, *P* = 0.001), N32 (*r* = 0.58, *P* = 0.002) and P50 amplitudes (*r* = 0.55, *P* = 0.003). For mastoid stimulation, the magnitude of the post response inhibition was correlated with the P12 (*r* = 0.59, *P* < 0.001) and N17 peak amplitudes (*r* = 0.46, *P* = 0.005). For sternal stimulation, the magnitude of the inhibition was significantly correlated with the P21 peak amplitude (*r* = 0.62, *P* < 0.001).

## Discussion

We have previously reported the properties of the potentials evoked over the posterior skull by stimuli that are known to activate otolith afferents, specifically impulsive stimuli, 500 Hz bone-conducted vibration and loud air-conducted sounds (Govender et al. 2020). We were following up earlier reports which had indicated the presence of a cerebellar contribution to these potentials, as shown with brain electrical source analysis (BESA: Todd et al. 2008b, 2017). We argued at the time that the short latency positive–negative response (P12–N17) was likely to be generated by the cerebellum, and, given the response latency, polarity, laterality and magnitude, presumably by Purkinje neurons responding to climbing fibre (CF) inputs (Todd et al. 2018b). CF responses to somatosensory stimulation characteristically occur with a latency of 6–15 ms with a surface positive polarity and the olivary-cerebellar tract from which they arise is strictly crossed (Eccles et al. 1968). The presence of post CEP pausing in the ECeG is also consistent with a CF interpretation (Latham and Paul 1971).

Since our report of the effects of mastoid stimuli (Govender et al. 2020), we have also examined axial stimuli for the possibility of recording associated CEPs over the posterior fossa (Todd et al. 2021). Axial stimuli share with vestibular stimuli the ability to evoke postural reflexes but do not depend on vestibular afferents, as postural reflexes to axial stimuli are preserved despite vestibular impairment (Bötzel et al. 2001; Graus et al. 2013). We confirmed here that these stimuli (C7 and sternal) also evoke short latency responses in electrodes overlying the cerebellum, but with a differing localisation and latency to the vestibular-evoked responses. We also found that subjects with large CEPs to one stimulus tended to have large responses to all. Source analysis has confirmed that the major contribution to these early potentials lies within the cerebellum, in the midline for the axial stimulus and slightly more lateral for the vestibular-evoked projections (Todd et al. 2021). Both vestibulo- and spino-olivary pathways are well-described (Brodal 1981; Barmack and Yakhnista 2015).

Bickford et al. (1964) reported myogenic potentials in neck muscles in response to a variety of stimuli and it is important to rule out a myogenic origin for the potentials we have been studying here. The vestibular-evoked myogenic potential (VEMP), probably the best known of these myogenic potentials, requires a significant level of background contraction to be detectable (Rosengren 2015). Govender et al. (2020) recorded their patients lying supine to ensure that the neck muscles were relaxed and thus unlikely to generate myogenic responses. In contrast, Todd et al.’s (2021) subjects were recorded while standing, but no neck sources appeared among the four major source locations despite this. The risk of myogenic contamination may thus be low with normal levels of tonic neck muscle activation.

As mentioned in the Introduction, gating of postural reflexes by task is one of their fundamental properties. The initial components at least are likely to be mediated through the brainstem and its descending tracts (Britton et al. 1993; Teng et al. 2017). This gating does not occur at the level of the motoneurone as tonic activation of the target muscles is not sufficient to overcome it, as shown here and previously (Britton et al. 1993; Govender et al. 2015). The muscles must be actively involved in the maintenance of balance for a response to be present and the response targets the most relevant muscle group, the “subconscious motor system” of Marsden et al. (1981). In the present study, the CEP showed no clear dependence upon posture, being present for all three stimuli both when lying and standing. There was higher tonic activity and responses when prone but not while supine, suggesting this was due to a change in the relative electrode positions with the head flexed, rather than being related to the posture itself. The lack of change of the CEP with posture indicates that gating at the afferent level, at least of the presumed spino-olivary input, is not primarily responsible for the attenuation of the short latency EMG reflexes when lying. If the major gating does not act at the afferent input or the spinal level, it follows that the modulation may occur at the level of the brainstem, quite plausibly by cortico-reticular projections. This would explain gating of postural reflexes by the cortical projection providing facilitation to reticular neurons when standing, thereby facilitating postural reflexes. The cortico-reticular tract arises from motor and premotor cortices, projects to the pontine reticular formation and the ponto-medullary reticular formation and innervates proximal muscles (Jang and Lee 2019). It is known to be involved in gait and postural stability (Matsuyama et al. 2004; Jang and Lee 2019).

Although the major basis for gating of postural reflexes does not appear to reside with the afferent input, there was a correlation between the CEP amplitude and the evoked reflexes for C7 stimuli when subjects were standing with anterior vs posterior lean. We have previously reported that forward lean increases the reflex evoked by C7 stimulation, by more than can be explained by the change in tonic activity (Govender et al. 2015). This observation in turn suggests that, once the brainstem is facilitated, changes in afferent feedback might modulate the size of the evoked reflexes. Unfortunately our results are not entirely consistent with this interpretation, given that sternal stimulation also gave a trend for larger responses for anterior lean, a situation in which the evoked EMG response was much less. While this may indicate that the afferent input is simply increased by leaning forwards, the sternal responses evoked were small and the findings may not be completely representative.

While we have confirmed the post-discharge inhibition characteristic of Purkinje cell CF responses for axial stimuli, previously shown following vestibular stimulation (Govender et al. 2020; Todd et al. 2021), these probably do not underlie the EMG reflex responses through cerebellar disinhibition of the brainstem, as proposed by Eccles et al. (1975). While the onset of inhibition of background ECeG activity was early enough to contribute to the EMG responses, there were inconsistencies. For example, despite mastoid stimulation evoking the most profound inhibition of ECeG activity (Fig. 8), it evoked the smallest reflexes in soleus. Both C7 and sternal stimuli evoked similar duration postural reflexes in opposing muscles, but their ECeG inhibitory profiles were different. It is important to note, however, that we were necessarily recording from a population of discharges and that this may not accurately reflect the activity of subpopulations of Purkinje cells with specific targets. The evidence from clinical observations suggests that the cerebellum modulates or shapes postural responses rather than primarily generating them (Horak and Diener 1994; Timmann and Horak 1997). Purkinje cell complex spikes are known to be generated in a number of other contexts, including by unexpected perturbations (Andersson and Armstrong 1987) and the activity we have recorded is also consistent with a response to perturbations of posture.

## Data Availability

Anonymised datasets are available from the corresponding author on reasonable request.
